# A Red-Emitting Cu(I)–Halide Cluster Phosphor with Near-Unity Photoluminescence Efficiency for High-Power wLED Applications

**DOI:** 10.3390/molecules27144441

**Published:** 2022-07-11

**Authors:** Wenjiang Zhaxi, Miao Li, Jing Wu, Luying Liu, Zetao Huang, Huixian Miao, Xiao Ma, Shenlong Jiang, Qun Zhang, Wei Huang, Dayu Wu

**Affiliations:** 1 Jiangsu Key Laboratory of Advanced Catalytic Materials and Technology, Advanced Catalysis & Green Manufacturing Collaborative Innovation Center, School of Petrochemical Engineering, Changzhou University, Changzhou 213164, China; 20070301003@smail.cczu.edu.cn (W.Z.); 19070303220@smail.cczu.edu.cn (M.L.); 19085216335@smail.cczu.edu.cn (J.W.); 20085600204@smail.cczu.edu.cn (L.L.); 20085600200@smail.cczu.edu.cn (Z.H.); 20085600356@smail.cczu.edu.cn (H.M.); maxiao@cczu.edu.cn (X.M.); 2 Hefei National Research Center for Physical Sciences at the Microscale, Department of Chemical Physics, Synergetic Innovation Center of Quantum Information & Quantum Physics, University of Science and Technology of China, Hefei 230026, China; poetjsl@ustc.edu.cn (S.J.); qunzh@ustc.edu.cn (Q.Z.)

**Keywords:** Cu(I)–halide cluster, photoluminescence, light-emitting diodes, phosphor-converted white LEDs

## Abstract

Solid-state lighting technology, where light-emitting diodes (LEDs) are used for energy conversion from electricity to light, is considered a next-generation lighting technology. One of the significant challenges in the field is the synthesis of high-efficiency phosphors for designing phosphor-converted white LEDs under high flux operating currents. Here, we reported the synthesis, structure, and photophysical properties of a tetranuclear Cu(I)–halide cluster phosphor, **[bppmCu_2_I_2_]_2_** (bppm = bisdiphenylphosphinemethane), for the fabrication of high-performance white LEDs. The PL investigations demonstrated that the red emission exhibits a near-unity photoluminescence quantum yield at room temperature and unusual spectral broadening with increasing temperature in the crystalline state. Considering the excellent photophysical properties, the crystalline sample of **[bppmCu_2_I_2_]_2_** was successfully applied for the fabrication of phosphor-converted white LEDs. The prototype white LED device exhibited a continuous rise in brightness in the range of a high bias current (100–1000 mA) with CRI as high as 84 and CCT of 5828 K, implying great potential for high-quality white LEDs.

## 1. Introduction

Highly luminescent molecules or clusters have attracted much attention in the past two decades because of their wide applications in fields of display, sensing, light-emitting devices, etc. [1,2,3,4,5,6]. The photophysical properties of metal complexes can be modified not only by the property of central metals but also by organic ligands and metal–ligand interactions [7,8,9,10,11,12,13,14,15,16,17]. Of particular interest is the potential use of Cu(I) complexes for light generation via electroluminescence [18,19,20,21,22,23,24,25], such as organic light-emitting diodes (oLEDs), because of their low cost, low toxicity, and earth-abundance of the copper element. Over the past two decades, luminescent Cu(I) complexes have been widely investigated as emitters in lighting devices [26,27,28,29,30]. However, the Cu(I) complexes, especially with multicenter cluster structure, usually exhibit weak room-temperature phosphorescence from either metal–ligand charge transfer (^3^MLCT) and/or cluster-centered (^3^CC) triplet excited states induced by moderate spin-orbit coupling (SOC) [31,32,33,34,35,36,37,38,39,40,41,42,43,44,45]. The structural distortions in the excited state enhance the probability of non-radiative processes, giving rise to poor photoluminescence quantum yield (PLQY). Hence, it is crucial to minimize structural distortion of local coordination spheres of metal centers for achieving highly emissive excited states [46,47,48,49]. We have previously demonstrated the synthesis and photophysical properties of cluster-based extended Cu(I) frameworks through monochelating imidazole derivatives, which showed negative/zero thermal quenching via electronic structural transition [15,16]. However, the monochelating metal complexes readily suffer from the heavy distortion of their excited states, which results in rather low PL efficiency, and the complexes are not attractive for LED phosphors. Therefore, we decided to synthesize related metal complexes that exhibit high PL quantum yields for potential applications.

**Scheme 1:**
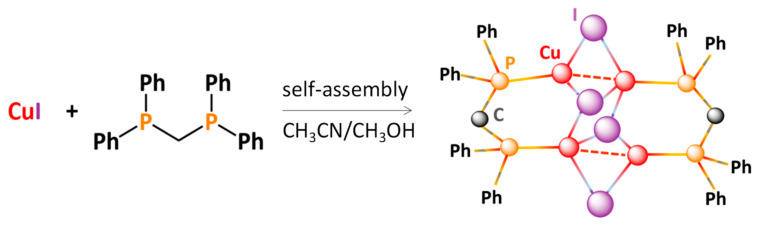
Synthetic method of the Cu(I)–halide cluster.

In this work, we report the synthesis, structure and application of a red-emitting tetranuclear Cu(I)–halide cluster phosphor with the formula of **[bppmCu_2_I_2_]_2_** from a bidentate organophosphate ligand, bisdiphenylphosphinemethane (bppm). The chemical structure of the bppm ligand and the formation of its complex studied here are shown in Scheme 1. The bischelating nature of ligand leads us to propose the cluster structure rather than an extended framework, in which the structural distortions are strongly reduced by introducing multichelating donors and sterical hindrances, such as the phenyl groups to the phosphine ligand (Scheme 1). This synthetic strategy is highly successful related to its PLQY at room temperature. We found that the tetranuclear copper cluster exhibited a broadband red emission with impressively high PL quantum yield (PLQY) exceeding 98% at room temperature in the crystalline state. The photophysical properties of the complex, **[bppmCu_2_I_2_]_2_**, are discussed with reference to the crystal structure, femtosecond transient absorption (fs-TA), and temperature-dependent PL emission. Furthermore, when the title Cu(I)–halide cluster was applied as phosphor for fabricating phosphor-converted white LEDs (pc-wLEDs), the pc-wLED devices showed a continuous brightness increase even under high flux working current owing to its high PL efficiency and photostability.

## 2. Results and Discussion

### 2.1. Structural Analysis

**Figure 1:**
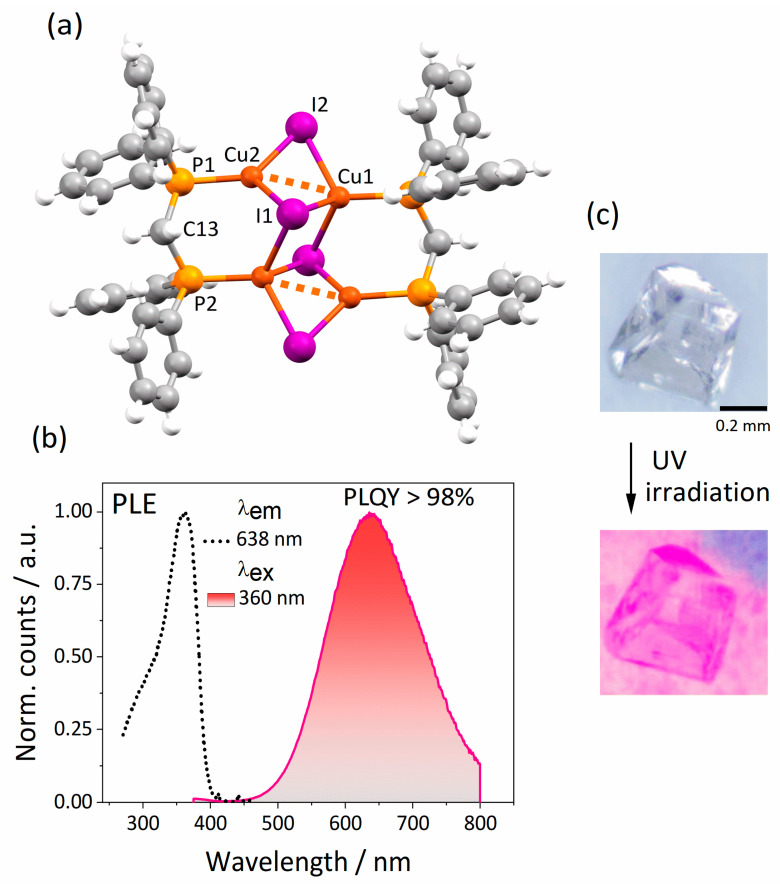
(**a**) Crystal structure of **[bppmCu_2_I_2_]_2_** at 100 K. Cu: red, C: gray, P: yellow, I: purple, H: white. (**b**) The normalized emission (λ_ex_ = 360 nm) and excitation (λ_em_ = 638 nm) spectra of **[bppmCu_2_I_2_]_2_** at room temperature. (**c**) Photographs of a 0.45 × 0.40 × 0.22 mm single crystal of **[bppmCu_2_I_2_]_2_**, under natural light and 365 nm UV irradiation, respectively.

The reaction between the bidentate organophosphate ligand (bppm) and CuI at room temperature gave rise to the self-assembly tetranuclear cluster with formula of **[bppmCu_2_I_2_]_2_**. The colorless bulk crystals of **[bppmCu_2_I_2_]_2_** were obtained through evaporating the resulting solution in the dark. The phase purity of the single-crystal sample is verified through powder XRD pattern (SI, Figure S1) and elemental microanalysis. The analysis of single-crystal X-ray diffraction data at 100 K indicated that the **[bppmCu_2_I_2_]_2_** cluster crystallized in the orthorhombic *P_bca_* space group and the asymmetric unit contained crystallographically distinct Cu_2_I_2_ rhomboid and a bppm ligand (Figure 1a and Table S1). It can be seen from Figure 1a that the centrosymmetric Cu_4_I_4_ unit forms an open chair structure. It is noticed that the Cu1–Cu2 distance (2.7309 Å) is short compared to the sum of the van der Waals radii of copper atoms (2.8 Å), which is indicative of a significant Cu–Cu interaction in the complex [50]. Given the bischelating character of bisphosphine ligand and cuprophilic interactions, the crystalline sample shows high thermal stability and begins to collapse above 400 °C (SI, Figure S2).

### 2.2. Photoluminescence Properties

**Scheme 2:**
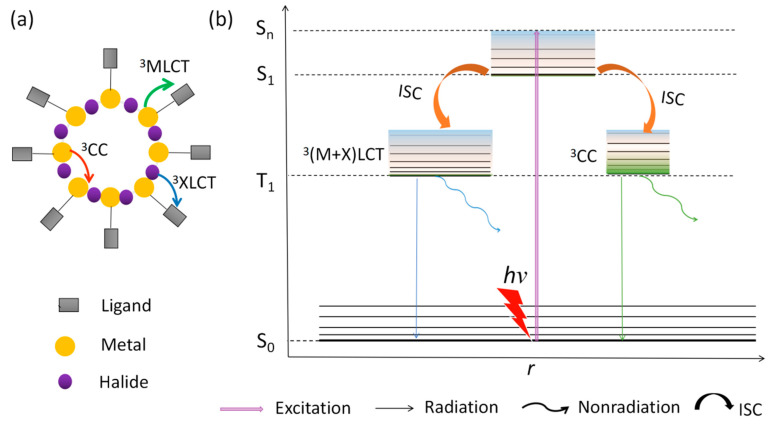
(**a**) Schematic structure and excited-state transitions of metal–halide clusters. M is metal; L is ligand; X is halide. (**b**) Excited-state decay pathways of Cu–halide clusters. ^3^(M+X)LCT, mixed metal– and halide–ligand charger transfer transition; ^3^CC, cluster-centered; ISC, intersystem crossing; Superscript‘3‘refers to triplet excited state.

The fluorescence spectrum of complex **[bppmCu_2_I_2_]_2_** at 298 K shows a wide red emission with a center peak at 638 nm and a half-peak width (FWHM) of 140 nm under the excitation wavelength at 360 nm (Figure 1b,c). The complex exhibits a large Stokes shift of 278 nm, indicating a significant energy level difference between the absorption excitation state and PL emission state. Notably, the high photoluminescence quantum yield is recorded to be 98.2% (SI, Figure S3). As is known, the photoluminescence properties of Cu(I)–halide complexes typically arise from two separate emissive states, as shown in Scheme 2. A low-energy emission, i.e., λ_em_ > 550 nm, which is assigned to the cluster-centered (^3^CC) excited state can be observed for systems with intermetallic interactions and is independent of the nature of the ligands. In addition, a high-energy emission, i.e., λ_em_ < 450 nm, is typically observed at low temperature, which is attributed to a ^3^(M+X)LCT excited state [2,4,5,15,18]. However, the high-energy emission is not observed even at the lowest temperature (79 K) in this case. Hence, we propose a qualitative model for the luminescence origin of **[bppmCu_2_I_2_]_2_** based on the above experimental data and the existing model [24,25,31,32,33,34,35]. The luminescence of **[bppmCu_2_I_2_]_2_** can be tentatively attributed to the ^3^CC excited state with a low-energy emission at 638 nm.

**Figure 2:**
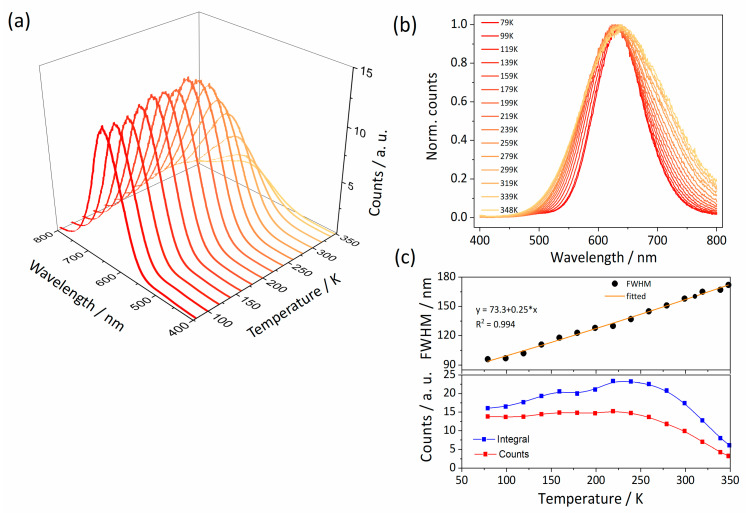
(**a**) PL emission spectra of **[bppmCu_2_I_2_]_2_** sample at temperatures from 77 to 350 K with λ_ex_ = 360 nm. (**b**) Normalized emission spectra of **[bppmCu_2_I_2_]_2_** at different temperatures. (**c**) Temperature-dependent FWHM (top) and PL intensity (bottom).

Upon heating the sample at low temperature, the cluster of **[bppmCu_2_I_2_]_2_** exhibits a thermally stable emission, with slight emission variation up to a temperature of 239 K, and the PL decreases upon further warming (Figure 2a). It can be seen from Figure 2b that the FWHM of the complex evidenced significant broadening as the temperature rise. Temperature dependence of FWHM of the emission peak is shown in Figure 2c (top), which indicates that the broadband red emission from **[bppmCu_2_I_2_]_2_** likely originates from distorted excited states (ES) due to the weakening Cu–Cu interactions at high temperature. The temperature dependence of the FWHM is well approximately fitted with a linear equation, and the PL emission increases with decreasing temperature and reaches its plateau at 239 K [51,52] (Figure 2c (below)).

### 2.3. Excited State Analysis

**Figure 3:**
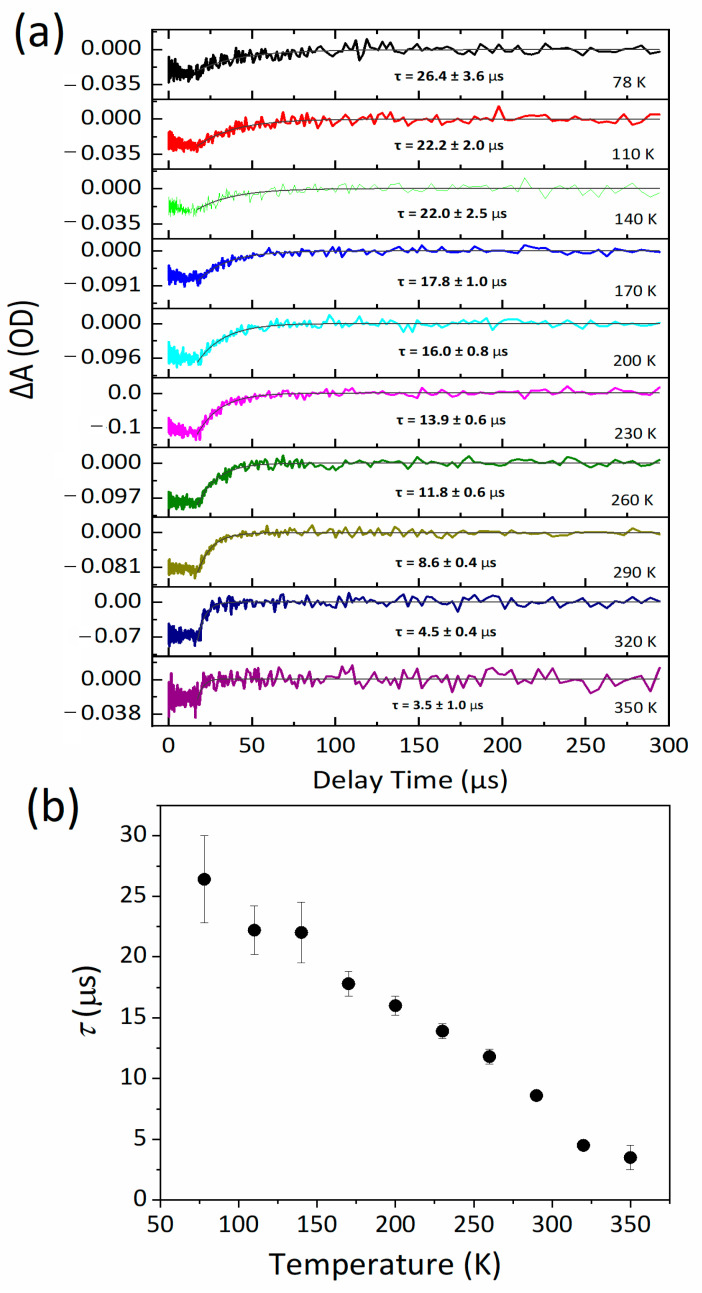
(**a**) The kinetic traces of **[bppmCu_2_I_2_]_2_** taken at each peak of the spectral profile at different temperatures. (**b**) Temperature dependence of the decay times for the complex **[bppmCu_2_I_2_]_2_**.

To deeply investigate the properties of excited states, we performed the femtosecond time-resolved transient absorption (fs-TA) spectroscopy from low to high temperature. Based on the PL emission procedure, the excitation light induces S_1_ excitation, and the essentially same spectral profiles for samples at different temperatures were obtained through the white-light continuum probe (400–800 nm) (SI, Figure S4). The temperature dependence of fs-TA echoes with those of fluorescence spectra. As shown in Figure 3a, the analysis of the fs-TA kinetics demonstrates the excited-state electronic transitions at different temperatures [53]. The exponential decay lifetime (i.e., 8.6 μs at 290 K) after equilibrium is consistent with the data through PL decay analysis (SI, Figure S5). Given the emission arises from thermally induced distorted ES, the PL decay times would be expected to increase as the distortion of these ES would be reduced at low temperatures. To further investigate the nature of excited states, temperature dependence of decay times were examined in the temperature range of 77–350 K as shown in Figure 3a. The PL decay times increase linearly upon decreasing temperature from 350 to 78 K (Figure 3b). Strong coupling of excited electrons with local structural distortion can result in a wide PL spectrum with a large Stokes shift due to distorted ES with respect to the ground state (GS).

### 2.4. LED Performance

**Figure 4:**
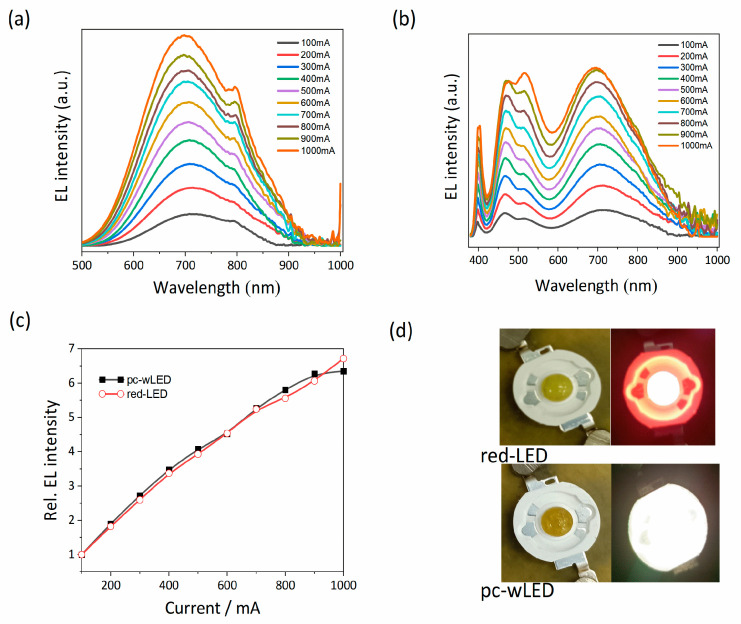
(**a**) Electroluminescence spectra of **[bppmCu_2_I_2_]_2_**-based single-component LED at high flux operating currents of 100–1000 mA. (**b**) Electroluminescence spectra of **[bppmCu_2_I_2_]_2_**-based pc-wLEDs at flux operating currents of 100–1000 mA. (**c**) Current-dependent EL intensities of single-component and its two-component wLEDs. (**d**) Image of **[bppmCu_2_I_2_]_2_**-based LED and pc-wLED prototype at a flux current of 1000 mA.

Initially, we investigated the high-power electroluminescence (EL) performance of **[bppmCu_2_I_2_]_2_** phosphor with UV-LED chips (λ_max_ = 365 nm). The results for the single-component red emission are shown in Figure 4a. The EL intensities of the red-emitting **[bppmCu_2_I_2_]_2_** phosphor increase as the current increases from 100 to 1000 mA (Figure 4a) [54,55]. According to the Commission Internationale d’Eclairage (CIE) chromaticity diagram, the corresponding CIE coordinates slightly change from (0.59, 0.31) at 100 mA to (0.55, 0.34) at 1000 mA, which is close to a standard red emitter with the CIE coordinates of (0.66, 0.33) (SI, Table S3).

In order to investigate the utility of **[bppmCu_2_I_2_]_2_** as a wideband red emitter for white LEDs, we further prepared phosphor-converted white LEDs with various correlated colour temperatures (CCTs). Exemplarily, the phosphor-converted white LED comprises a commercially available blue-emitting BAM:Eu^2+^ (465 nm, 58%), green emitting phosphor (Ba,Sr)_2_SiO_4_:Eu^2+^ (515 nm, 19%), and **[bppmCu_2_I_2_]_2_** crystalline sample (23%). For **[bppmCu_2_I_2_]_2_**-based pc-wLED, the whole visible region from 400 to 750 nm was obtained in the EL spectra (i.e., a white-light emission), which increased under the high flux operating currents (Figure 4b). Interestingly, the **[bppmCu_2_I_2_]_2_**-based single-component and pc-wLED exhibited a similar linear increase in intensity with increasing operating current, demonstrating excellent photostability (Figure 4c). To further explore the potential of practical applications as pc-wLED devices, the color stability for high-power LED operation was studied. The CIE *x* (ca. 0.32) of **[bppmCu_2_I_2_]_2_**-based wLED is nearly constant, and CIE *y* exhibited a small change (from 0.32 to 0.35) upon lifting the flux current from 100 to 1000 mA (SI, Table S4). The CRI value of **[bppmCu_2_I_2_]_2_**-based wLED was 84 and a CCT was 5828 K at a flux current of 1000 mA at 3.8 V (Figure 4d). Hence, the complex **[bppmCu_2_I_2_]_2_** shows excellent performance among the high efficient phosphors for designing high-power white LED devices (Table 1) [56].

## 3. Materials and Method

### 3.1. Materials

The chemicals and reagents, including bis(diphenylphosphino)methane (bppm) and copper(I) iodide, were commercially obtained and used as received without further purification. LED chips for designing the red and white electroluminescence devices were purchased from San‘an Optoelectronics Co., Ltd (Xiamen, China).

### 3.2. Synthesis of **[bppmCu_2_I_2_]_2_** Cluster

To a 10 mL stirred methanol solution containing bis(diphenylphosphino)methane (19.22 mg, 0.05 mmol) was added CuI (19.04 mg, 0.1 mmol) dissolved in 10 mL of acetonitrile solution, which was magnetically stirred for another 10 min. The block colorless crystals suitable for X-ray diffraction analysis were obtained by evaporating the solution at room temperature for a week. Yield: 65%. Infrared (IR) analysis (KBr, cm^−1^): 1950(w), 1895(w), 1817(w), 1774(w), 1648(m), 1585(m), 1572(s), 1432(s), 1343(s), 1369(m), 1309(m), 1276(w), 1185(s), 1157(m), 1137(m), 1098(s), 1070(w), 1026(s), 1000(s), 909(w), 838(w), 774(m), 737(m), 719(w), 689(m), 617(w), 519(s), 469(m), 445(w), 419(w). Anal. Calcd. for C_25_H_22_Cu_2_I_2_P_2_: C, 38.56; H, 2.90. Found: C, 39.20; H, 2.87.

### 3.3. Spectroscopic Measurements

An Edinburgh FS5 model instrument was used to record fluorescence spectra of **[bppmCu_2_I_2_]_2_** with the slit width at 0.5 nm for both excitation and emission in all the experiments. An Edinburgh QY system, equipped with an integrating sphere was available to record the absolute PL quantum yield (PLQY) at room temperature. An Edinburgh FLS980 steady-state fluorimeter was used to analyze PL decays through a time-correlated single-photon counting spectrometer. A Nicolet Magna-IR750 spectrophotometer with the spectral range of 4000−400 cm^−1^ was available to record the FT-IR spectra of **[bppmCu_2_I_2_]_2_** in KBr pallet (w, weak; b, broad; m, medium; s, strong). A RINT2000 vertical goniometer equipped with CuK_α_ X-ray source (operated at 40 kV and 100 mA) was used to record powder X-ray diffraction (PXRD). Elemental analyses (C, H) were conducted with a Perkin Elmer 2400 analyzer.

### 3.4. Temperature-Dependent Ultrafast Transient Absorption Measurements

An EOS pump–probe system (Ultrafast Systems LLC., Sarasota, FL, USA) equipped with an amplified femtosecond laser system (Coherent) was available to perform the femtosecond time-resolved transient absorption (fs-TA) spectra. An optical parametric amplifier (TOPAS-800-fs) delivered the pump pulses (~56 μW), with a Ti:sapphire regenerative amplifier and a mode-locked Ti:sapphire laser system (Micra 5). A picosecond Nd:YAG laser beam generates the WLC probe pulses (400–800 nm) into a photonic crystal fiber. Between the pump and probe pulses, the time delays in the range of 8 ns–100 μs are recorded through a digital delay generator. The IRF is determined to be ~100 ps. An optical fiber-coupled multichannel spectrometer with a CMOS sensor was used for analyzing the temporal and chirp-corrected spectral profiles of the pump-induced differential transmission of the WLC probe light. The temperature in the range of 77–350 K was controlled through an Oxford cryogenic system.

### 3.5. Crystal Structure Determination

A Bruker APEX-2 CCD with graphite-monochromated MoK_α_ radiation (λ = 0.71073 Å) was used for collecting the diffraction intensity data of **[bppmCu_2_I_2_]_2_** at 100 K. The Bruker Instrument Service v4.2.2 and SAINTV 8.34A software were used to perform data collection, data reduction, and cell refinement [63,64]. The structural analyses were performed with direct methods through the SHELXS program, and the full-matrix least-squares routine was used for refinement with SHELXL based on *F*^2^ [65]. Multiscan program SADABS software was available to perform absorption corrections [66]. The C, P, Cu, and I atoms were anisotropically refined with the SHELXTL program package, and hydrogen atoms on organic ligands were geometrically generated by the riding mode [67,68].

CCDC no. 2179774. The crystallographic data can be obtained free of charge via www.ccdc.cam.ac.uk/conts/retrieving.html (accessed on 28 June 2022) (or from the Cambridge Crystallographic Data Centre, 12 Union Road, Cambridge CB2 1EZ, UK).

### 3.6. TGA Analysis

The Labsys Evo thermal gravimetric analyzer was used for thermogravimetric (TGA) analyses of **[bppmCu_2_I_2_]_2_** under the atmosphere of N_2_. The crystalline sample (ca. 5 mg) was placed on a platinum pan and the data was performed in the range from room temperature to 900 °C at a rate of 10 °C/min.

### 3.7. LED Devices Fabrication

The stoichiometric **[bppmCu_2_I_2_]_2_** and pouring sealant (ZWL8820) were mixed and stirred for 10 min, the mixture was then deaerated in vacuum oven at room temperature. A fully packaged Epileds InGaN LED chip (an emission wavelength of ca. 360 nm) was covered by the mixture with the forward bias current of the LEDs in the range of 100–1000 mA. An Everfine HAAS-2000 equipment was available to record electroluminescence (EL) spectra of the LEDs at room temperature [69,70]. In all the measurements, an integrating sphere with a diameter of 30 cm was coupled to a high-accuracy array spectroradiometer (wavelength accuracy < 0.3 nm) and a programmable test power LED 300E [71]. For fabrication of white pc-LED, a UV LED chip was integrated with commercial blue phosphor (BAM:Eu^2+^), green phosphor (Ba,Sr)_2_SiO_4_:Eu^2+^ and **[bppmCu_2_I_2_]_2_** phosphor with the weight ratio of ca. 3:1:1.2.

## 4. Conclusions

In summary, a tetranuclear copper(I)-iodide cluster phosphor with a defined crystal structure exhibiting intense red photoluminescence has been prepared by using a bulky bidentate organophosphate ligand. The photophysical investigations indicate that the cluster phosphor exhibited an efficient room-temperature phosphorescence with a near-unity PL quantum yield reaching 98%. The complex shows a thermally induced broadening of PL spectra with short emission decay times (3.5–26 μs). Owing to the enhanced efficiency and stability, the title cluster can be considered a promising phosphor material. These results demonstrate an idea example for preparing highly efficient and photostable metal–halide phosphor with short decay times which are necessary for high-performance electroluminescence devices.

## Figures and Tables

**Table 1:** The performance of related Cu(I) complexes for LED applications.

Compound	λ_ex_ (nm)	λ_em_ (nm)	*I* (mA)	CRI	CCT (K)	CIE (*x*,*y*)	PLQY (%)	Reference
(18-crown-6)_2_Na_2_(H_2_O)_3_Cu_4_I_6_	450	536	20	89.6	5859	0.32, 0.34	91.8	[57]
(C_12_H_24_O_6_)CsCu_2_Br_3_	365	535	20–300	73.7	4962	0.34, 0.43	78.3	[58]
[Cu(PPh_3_)_2_(PmH)]BF_4_	395	537	20–120	50.8	4881	0.37, 0.52	26.0	[59]
[Cu(DPEphos) (PmH)]BF_4_	395	560	20–120	50.5	3737	0.44, 0.52	42.0	
[Cu(Xantphos) (PmH)]BF_4_	395	570	20–120	65.8	3561	0.44, 0.49	1.0	
Cu(PPh_3_)_2_(Pm)	395	550	20–120	62.1	4176	0.40, 0.50	27.0	
Cu(DPEphos)(Pm)	395	555	20–120	56.6	3875	0.43, 0.52	25.0	
Cu(Xantphos)(Pm)	395	565	20–120	59.1	3603	0.44, 0.51	17.0	
Cu(dppb)(2,2′-biquinoline)]BF_4_	365	735	60–100	69.2	1053	0.59, 0.29	–	[60]
[Cu(dppb)(bbim)]BF_4_	365	538	20–120	38.2	4496	0.39, 0.55	23.0	[61]
[Cu(DPEphos)(bbim)]BF_4_	365	533	20–120	54.4	5381	0.34, 0.50	12.0	
[Cu_3_(µ_3_-H)(µ_2_-dppy)_4_](ClO_4_)_2_	450	580–680	–	83.7	5281	0.33, 0.31	71.8	[62]
**[bppmCu_2_I_2_]_2_**	360	638	100–1000	84	5828	0.32, 0.34	98.2	This work

Note: PmH = 2-(pyridin-2-yl)benzimidazole. PPh3 = triphenylphosphine. DPEphos = bis(2-diphenylphosphinophenyl)ethe. Xantphos = 9, 9-dimethyl-bis(diphenylphosphino)xanthenes. dppb = 1, 2-bis(diphenyl phosphino)benzene, bbim = bisbenzimidazole. dppy = diphenyl-2-pyridylphosphine. bppm = bisdiphenylphosphinemethane. – not reported. The PLQY data are reported at room temperature.

## Data Availability

The data presented in this study are available in Supplementary Material.

## Supplementary Materials

The following supporting information can be downloaded at: https://www.mdpi.com/article/10.3390/molecules27144441/s1. Figure S1: The powder XRD patterns; Figure S2: TGA diagram; Figure S3: The measurements of PLQY; Figure S4: Temperature-dependent fs-TA spectra at the 1 μs probe delay. The fs-TA signal (that is, the absorbance changes) is given in OD which stands for optical density; Figure S5: PL decay curve at 298 K. Table S1: The crystallographic data and refinement parameters at 100K; Table S2: The selected bond distances (Å); Table S3: The EL parameters of **[****bppmCu_2_I_2_****]****_2_**-based LED; Table S4: The EL parameters of **[bppmCu_2_I_2_]_2_**-based pc-wLED.

## Sample Availability

Samples of the compounds are available from the authors upon request.
