# Supplementary material for: A Red-Emitting Cu(I)–Halide Cluster Phosphor with Near-Unity Photoluminescence Efficiency for High-Power wLED Applications
Source: Molecules. 2022 Jul 11;27(14):4441. doi: 10.3390/molecules27144441 (PMC9318059; doi:10.3390/molecules27144441)
Supplement: Supplementary file 1 [file molecules-27-04441-s001.zip › molecules-1816775-SI.pdf]

Supporting Information (SI) for

A Red-Emitting Cu(I)–Halide Cluster Phosphor with Near-Unity Photoluminescence Efficiency for High-Power wLED Applications

Wenjiang Zhaxi [1], Miao Li [1], Jing Wu [1], Luying Liu [1], Zetao Huang [1], Huixian Miao [1], Xiao Ma [1], Shenlong Jiang [2], Qun Zhang [2], Wei Huang [1,*] and Dayu Wu [1,*]

[1] Jiangsu Key Laboratory of Advanced Catalytic Materials and Technology, Advanced Catalysis & Green Manufacturing Collaborative Innovation Center, School of Petrochemical Engineering, Changzhou University, Changzhou 213164, China; e-mail@e-mail.com (W.Z.); 19070303220@smail.cczu.edu.cn (M.L.); 19085216335@smail.cczu.edu.cn (J.W.); 20085600204@smail.cczu.edu.cn (L.L.); 20085600200@smail.cczu.edu.cn (Z.H.); 20085600356@smail.cczu.edu.cn (H.M.); maxiao@cczu.edu.cn (X.M.)

[2] Hefei National Research Center for Physical Sciences at the Microscale, Department of Chemical Physics, Synergetic Innovation Center of Quantum Information & Quantum Physics, University of Science and Technology of China, Hefei 230026, China; poetjsl@ustc.edu.cn (S.J.); qunzh@ustc.edu.cn (Q.Z.)

* Correspondence: whuang@cczu.edu.cn (W.H.) or wudy@cczu.edu.cn (D.W.)

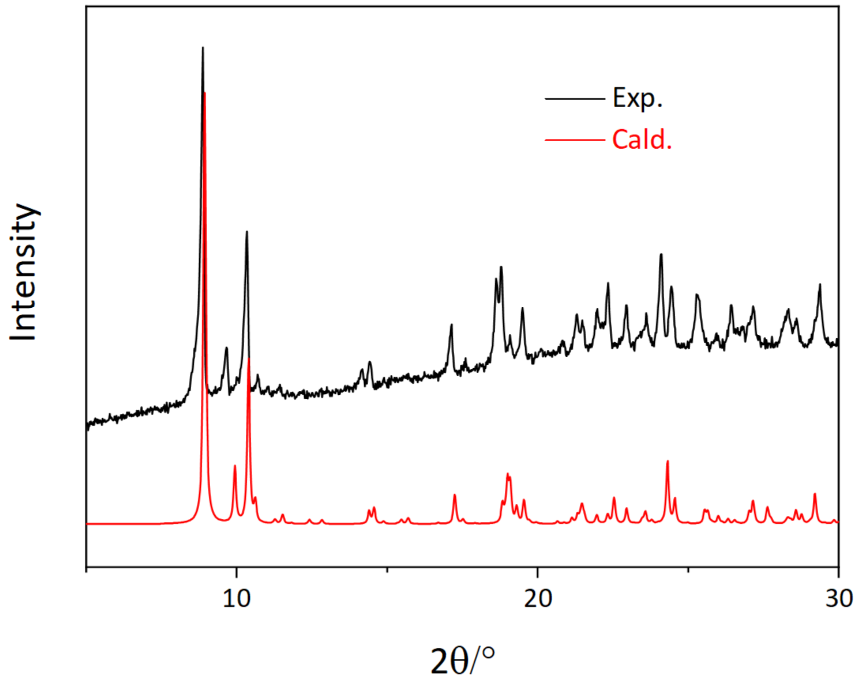

Figure S1. The powder XRD patterns of **[bppmCu$_2$I$_2$]$_2$**.

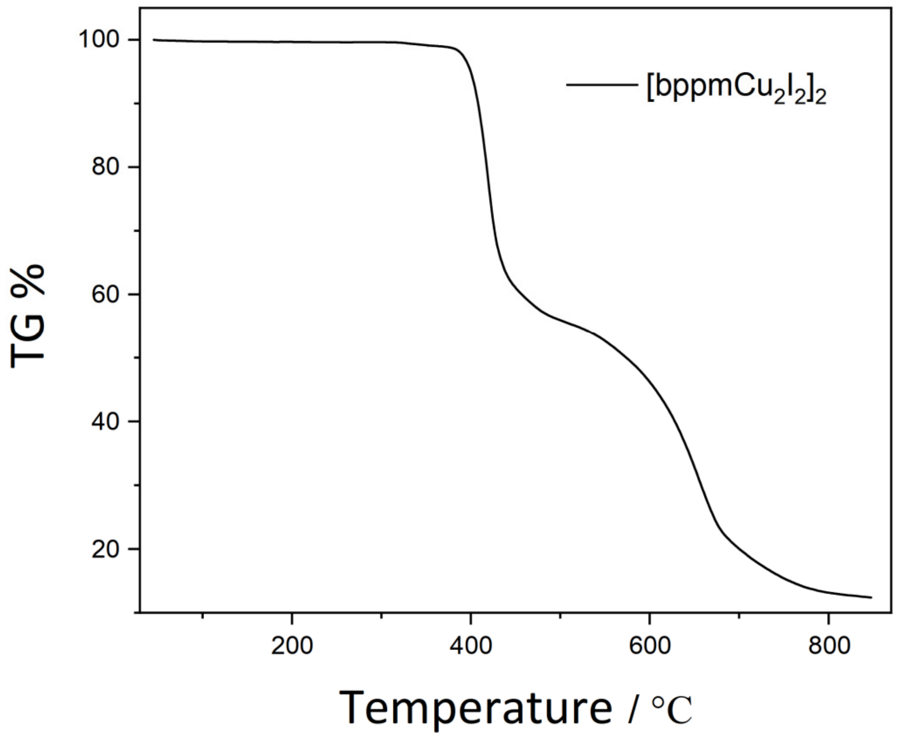

Figure S2. TGA diagram of **[bppmCu$_2$I$_2$]$_2$**.

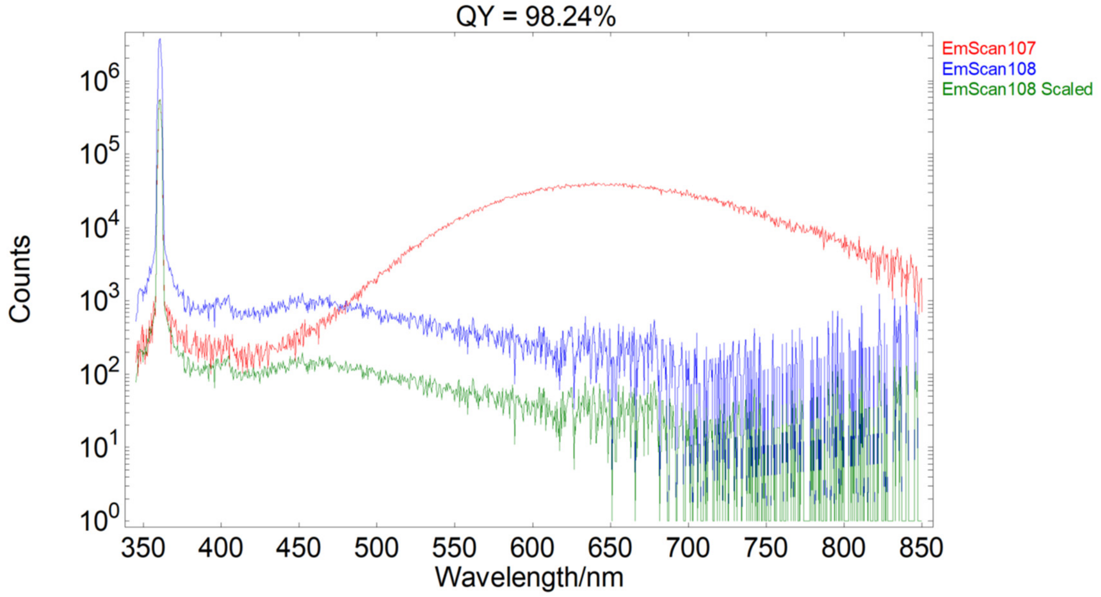

Figure S3. The measurements of PLQY for **[bppmCu$_2$I$_2$]$_2$**.

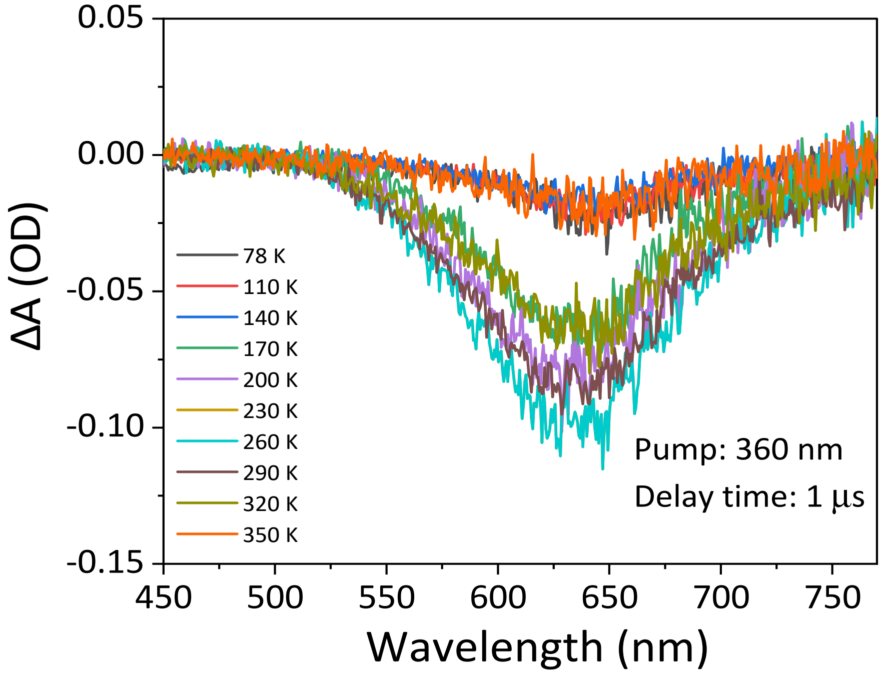

Figure S4. Temperature-dependent fs-TA spectra of **[bppmCu$_2$I$_2$]$_2$** at the 1 μs probe delay. The fs-TA signal (that is, the absorbance changes) is given in OD which stands for optical density.

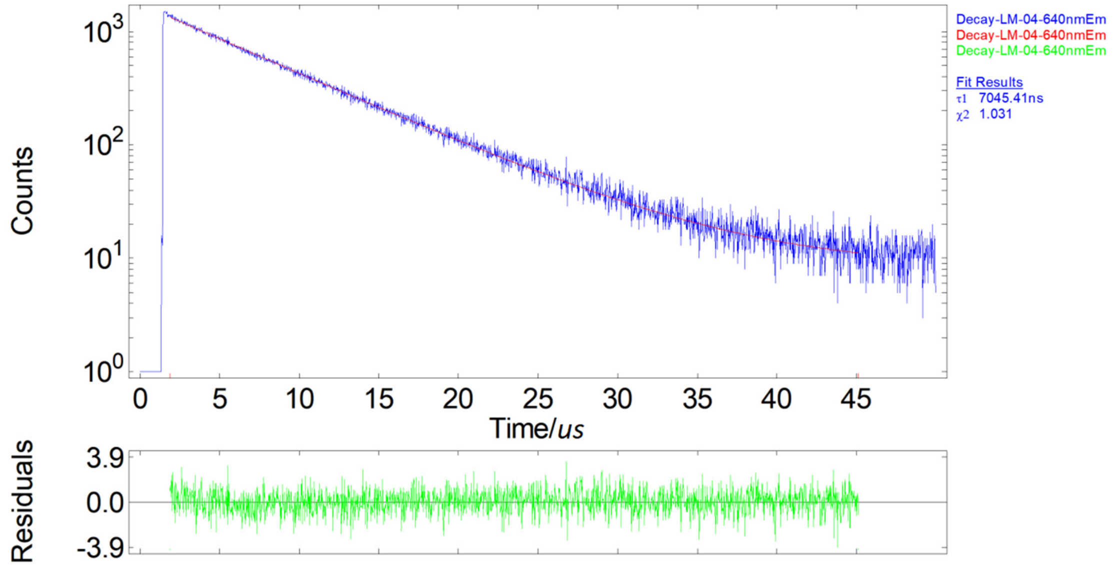

Figure S5. PL decay curve of **[bppmCu$_2$I$_2$]$_2$** at 298 K.

Table S1. The crystallographic data and refinement parameters at 100K.

Complex	$C_{25}H_{22}I_2Cu_2P_2$
Temperature (K)	100
Formula weight	765.24
Crystal system	orthorhombic
Space group	*Pbca*
a [Å]	16.6523(3)
b [Å]	17.0061(3)
c [Å]	17.7846(3)
α [º]	90
β [º]	90
γ [º]	90
V [Å^3]	5036.43(15)
Z	8
F (000)	2928
$R_1{}^a/wR_2{}^b[I>2\sigma(I)]$	0.0215/0.0447
R_1/wR_2 (*all date*)	0.0193/0.0439
GOF^c on F^2	1.043

[a] $R_1 = \sum||F_0|-|F_c||/\sum|F_0|$, [b] $wR_2 = \{\sum[w(F_o{}^2-F_c{}^2)^2]/\sum[w(F_0{}^2)^2]\}^{1/2}$.

[c] *Goodness-of-fit* = $[[w(F_0{}^2-F_c{}^2)^2]/(N_{obs}-N_{params})]^{1/2}$, based on the data $I>2\sigma(I)$.

Table S2. The selected bond distances (Å).

$C_{25}H_{22}Cu_2I_2P_2$	
Cu1 P2	2.2399(4)
Cu1 I2	2.6758(2)
Cu1 I1	2.6946(2)
Cu1 Cu2	2.7309(3)
Cu1 I1	2.7366(2)
Cu2 P1	2.2096(4)
Cu2 I2	2.5176(2)
Cu2 I1	2.6411(2)
P1 C5	1.8202(16)
P1 C12	1.8294(15)
P1 C13	1.8286(15)
P2 C18	1.8230(16)
P2 C24	1.8230(15)
P2 C13	1.8347(14)

Table S3. The EL parameters of **$[bppmCu_2I_2]_2$**-based LED.

Current [A]	0.1	0.2	0.3	0.4	0.5	0.6	0.7	0.8	0.9	1.0
Potential/V	3.04	3.12	3.2	3.27	3.34	3.42	3.5	3.57	3.65	3.74
CIE x	0.5888	0.5973	0.5879	0.5882	0.5752	0.5762	0.563	0.5572	0.544	0.5546
CIE y	0.3112	0.3373	0.3427	0.3407	0.3438	0.3403	0.3409	0.3418	0.3404	0.3416
R1	70.5	65.8	79	75.3	69.8	46.6	80.6	73.1	81.9	50.8
R2	89.8	79.3	92	89.5	82.4	58.8	92.8	85	93.5	62.4
R3	87.5	91.2	94.8	95.9	93.4	68.1	95.3	94.8	95.3	70.9
R4	54.9	83.2	69.9	67.6	83	90.6	71.6	82.3	73.4	91.1
R5	67.5	91.1	76.8	74.9	90.3	84.1	78.5	89.5	80.3	85.4
R6	87.6	92.6	90.3	89.2	95.1	69.7	90.3	96.6	91	72.2
R7	76.8	67.6	82.2	80	70.8	45.5	82.7	73.3	82.8	48.4
R8	63	55.1	69.8	66.1	59	32.8	71.7	62.6	72.9	36.8
R9	39.6	41.2	49.7	44.5	45	29.2	53	48.7	55.2	33.7
R10	84.1	68	84.9	80.6	72.1	39.9	85.6	75.5	86.7	44.8
R11	45	76.5	64	59.8	76.3	95.6	65.3	75.9	67.4	95.1
R12	79.6	88.7	79.7	75.2	85.6	88.6	76.6	82.4	75.4	86.8
R13	73.2	63.8	80.9	76.9	68.6	38.1	82.7	72.7	84.2	43.4
R14	94.8	91.2	98.4	98.5	93.3	76.5	98.4	94.5	98.3	78.7
R15	74.4	66.5	80.3	77.1	71	46.8	83	75	84.9	51.7
Ra	74.7	78.2	81.8	79.8	80.5	62	82.9	82.1	83.9	64.7
CCT [K]	1101	1184	1250	1238	1314	1292	1360	1394	1460	1408

Table S4. The EL parameters of **[bppmCu$_2$I$_2$]$_2$**-based pc-wLED.

Current/A	0.1	0.2	0.3	0.4	0.5	0.6	0.7	0.8	0.9	1.0
Potential/V	3.03	3.12	3.2	3.27	3.35	3.43	3.51	3.59	3.68	3.77
CIE x	0.297	0.3108	0.3156	0.3206	0.3207	0.3263	0.323	0.3255	0.3222	0.3245
CIE y	0.3188	0.3231	0.3234	0.3275	0.3282	0.3325	0.338	0.3442	0.3477	0.3564
R1	62.9	63.5	63.9	68.8	70.9	81.1	73.4	79.4	81.7	87.6
R2	73.6	75.6	76	79.7	81.6	90	82.2	86.1	87.6	96
R3	84.1	87.7	88.2	89.8	91.2	92.4	90	91.2	91.5	98.2
R4	84.7	79.3	76.6	74.7	76.1	70.5	83.1	83.1	88.3	90.4
R5	86.1	78.4	75.2	73.2	73.8	67.9	81.7	82.7	88.2	82.8
R6	86.9	81.6	78.9	77.3	75.9	69.9	81.8	84	86	74.4
R7	73.2	75.8	77.6	82.1	80.5	84.6	80.7	85.8	83.3	73.6
R8	43.5	45.2	46.9	53.7	55.9	67.8	60.1	68	69.7	71
R9	44.8	42.2	39.9	28.2	50.1	62.9	60.2	62.2	23.5	46.4
R10	45.3	47.2	47	52.7	56	68	59.7	66.8	70.8	86.1
R11	84.6	76.4	73.1	71.6	72.5	67.8	80.3	81.6	87.1	87.2
R12	82.3	76.7	72.9	71.5	72.2	68.9	78.9	81.1	86.7	89.2
R13	61.8	64	64.7	70.2	72.8	84.9	74.1	80.1	82.1	90.3
R14	90.2	92.6	93	93.7	94.8	94.9	94.2	94.7	95	98.8
R15	52.6	54.1	54.9	60.7	63.7	75.3	67.7	74.4	77.6	87.8
Ra	74.4	73.4	72.9	74.9	75.7	78	79.1	82.5	84.5	84.2
CCT [K]	7573	6664	6388	6092	6084	5789	5939	5806	5949	5828
